# miR-183-5p Is a Potential Molecular Marker of Systemic Lupus Erythematosus

**DOI:** 10.1155/2021/5547635

**Published:** 2021-05-06

**Authors:** Shaolan Zhou, Jing Zhang, Pengfei Luan, Zhanbing Ma, Jie Dang, Hong Zhu, Qian Ma, Yanfeng Wang, Zhenghao Huo

**Affiliations:** ^1^Department of Medical Genetics and Cell Biology, College of Basic Medicine, Ningxia Medical University, Yinchuan, Ningxia, China; ^2^Department of Rheumatology, General Hospital of Ningxia Medical University, Yinchuan, Ningxia, China; ^3^Key Laboratory of Fertility Preservation and Maintenance (Ningxia Medical University), Ministry of Education, Yinchuan, Ningxia, China; ^4^Ningxia Key Laboratory of Cerebrocranial Diseases, Ningxia Medical University, Yinchuan, Ningxia, China; ^5^Department of Biology, Gansu Medical College, Pingliang, Gansu, China

## Abstract

**Objective:**

To investigate microRNA (miRNA) expression profiles in individuals with systemic lupus erythematosus (SLE) and identify the valuable miRNA biomarkers in diagnosing and monitoring SLE.

**Methods:**

Next-generation sequencing (NGS) was performed to assess miRNA amounts in peripheral blood mononuclear cells (PBMCs) from four SLE cases and four healthy controls. Quantitative polymerase chain reaction (qPCR) was carried out for validating candidate miRNAs in 32 SLE cases and 32 healthy controls. In addition, receiver operating characteristic (ROC) curve analysis was completed to evaluate diagnostic performance. Finally, the associations of candidate miRNAs with various characteristics of SLE were analyzed.

**Results:**

A total of 157 miRNAs were upregulated, and 110 miRNAs were downregulated in PBMCs from SLE cases in comparison to healthy controls, of which the increase of miR-183-5p and decrease of miR-374b-3p were validated by qPCR and both showed good diagnostic performance for SLE diagnosis. Besides, miR-183-5p expression levels displayed a positive association with SLE disease activity index (SLEDAI) and anti-dsDNA antibody amounts.

**Conclusion:**

Our data indicated that miR-183-5p is a promising biomarker of SLE.

## 1. Introduction

Systemic lupus erythematosus (SLE) is an important chronic, inflammatory, and multisystem autoimmune pathology, featuring the production of autoantibodies against various nuclear self-antigens. The precise molecular mechanisms underlying the pathogenesis of SLE remain uncertain but encompass complex interactions of genetic, epigenetic, and environmental factors [1, 2]. Numerous reports have extensively focused on identifying susceptibility loci/genes in SLE [3–5]. Genome-wide association studies (GWAS) have revealed the major signaling pathways affected in SLE, but no single gene defect has been identified as the principal pathogenic factor contributing to SLE induction [6–8]. In addition, SLE monozygotic twins harboring identical genes show a low concordance in disease phenotypes, suggesting that nongenetic factors such as epigenetic parameters play a critical role in SLE pathogenesis [9, 10].

MicroRNAs (miRNAs) constitute a group of small, single-stranded noncoding RNAs, which suppress genes posttranscriptionally through binding to specific seed sequences in their target genes, causing translation inhibition or target gene degradation [11]. miRNA is a typical type of epigenetic modification, which contributes to the pathogeneses of multiple autoimmune diseases [12]. Previous evidence indicates the potential role of miRNAs in regulating immune cell development and maintaining immune homeostasis [13–16]. Vinuesa and collaborators identified multiple target sites for >140 conserved miRNAs in SLE susceptibility genes [17]. miRNAs are also known to have important functions in the molecular mechanisms of SLE by interacting with innate and adaptive immunity [18–21]. Furthermore, miRNAs represent potent biomarkers for the diagnosis and monitoring of diverse pathologies thanks to their high stability [22, 23]. However, the functions of miRNAs in the diagnosis and stratification of SLE remain undefined. Therefore, understanding the associations of miRNAs with SLE would provide novel insights into disease pathogenesis and help develop new diagnostic biomarkers [24].

The present study performed next-generation sequencing (NGS) to examine miRNA profiles in SLE cases and healthy subjects and to determine the values of select miRNAs in diagnosing and monitoring SLE.

## 2. Methods

### 2.1. Patients and Specimen Collection

In this study, SLE patients were enrolled in Rheumatology and Immunology Department, General Hospital of Ningxia Medical University. The subjects were included according to the criteria of the American College of Rheumatology (1997 revision) [25]. Subjects who had additional rheumatic pathologies, infectious diseases, or cancers were excluded. Disease activity was evaluated according to the SLE disease activity index-2000 (SLEDAI-2000) [26]. Age- and sex-matched healthy controls undergoing routine health exams were strictly assessed by two experienced rheumatologists and archived in parallel. All healthy controls with any medical histories (including rheumatic pathologies, infectious diseases, or cancers), family histories (including rheumatic pathologies), or rheumatic manifestations (including nephritis, arthritis, rash, and serositis) were excluded. The peripheral venous blood from each subject was collected in evacuated tubes containing EDTA as the anticoagulant and peripheral blood mononuclear cells (PBMCs) were isolated within 2 hours. This study was approved by the Ethics Committee of General Hospital of Ningxia Medical University. All participants signed written informed consent.

### 2.2. Study Flow

The study comprised two main phases, the exploration and validation. In the exploration phase, PBMC specimens from four SLE cases and four healthy controls were examined in NGS to detect miRNA expression profiles. In the validation phase, PBMC specimens from 32 SLE patients and 32 healthy controls were assessed by quantitative polymerase chain reaction (qPCR) to detect the expression levels of candidate miRNAs for NGS data validation.

### 2.3. Clinical and Laboratory Assessments

The features of SLE and control cases in the validation phase are displayed in Table 1. Clinical symptoms in SLE were lupus nephritis, arthritis (at least two joints involved), rash (including discoid or butterfly rash, oral ulcer, and photosensitivity), and serositis (including pleuritis and pericarditis). Laboratory features included erythrocyte sedimentation rate (ESR), hypersensitive C-reactive protein (CRP), complement 3 (C3), and anti-dsDNA antibody. The SLEDAI was assessed for each patient.

### 2.4. PBMC Isolation and RNA Purification

PBMC isolation was performed from SLE and healthy control cases by the Ficoll-Paque density gradient centrifugation assay. Briefly, the diluted blood sample was carefully layer on Ficoll-Paque and centrifuged at 400 g for 30 min at 18°C to 20°C, the layers of mononuclear cells were transferred to a sterile centrifuge tube, and the isolated cells were washed with balanced salt solution [27]. Total RNA was purified with Trizol by the protocol as provided by the manufacturer. RNA quality and amounts were evaluated on a NanoDrop 2000 (Thermo, USA), by 1% agarose gel electrophoresis and Agilent 2100 Bioanalyzer (Agilent Technologies, USA).

### 2.5. Library Generation and Sequencing

The miRNA sequencing libraries were prepared from extracted miRNA samples using the Illumina small RNA sample prep kit according to instructions provided by the manufacturer. Small RNAs (18–30 nt) were isolated from total RNA to perform ligations with 5′ and 3′ adapters. Then, RT-PCR was completed taking the ligation products as templates. Finally, the PCR products were clustered, and the Illumina HiSeq™2500 platform (Illumina, USA) was utilized for sequencing.

### 2.6. Differential Expression Analysis

For miRNA sequencing, sequence reads were cleaned after library construction. Then, miRNAs in various groups were compared after raw read count normalization; the data were log-transformed according to the fold change (FC) formula [FC = log2 (treatment/control)]. Statistical significance was defined as *p* < 0.05 and log2 (FC) > 0.5.

### 2.7. Preparation of cDNA and qPCR

Complementary DNA (cDNA) was synthesized with the PrimeScript™ RT reagent kit (Takara Biotech, China) with miRNA stem loop primers. The qPCR reactions were carried out with TB Green® Premix Ex Taq™ II kit (Takara Biotech) on an ABI 7500 real-time PCR system (Applied Biosystems, USA). The PCR amplification procedure was as follows: denaturation at 95°C for 30s, followed by 40 cycles of 95°C for 5 s and 34 s at 60°C. Candidate miRNA and mRNA amounts were calculated by the 2^-*ΔΔ*Ct^ method, with U6 and GAPDH used for normalization, respectively. Primers for qPCR are shown in Supplementary Table 1.

### 2.8. Dual-Luciferase Reporter Assay

To develop the plasmid containing human Foxo1 3′UTR, plasmid GV272 was purchased from Shanghai Genechem Company and digested with *Xba*I. In this plasmid, the expression of firefly luciferase is driven by SV40 promoter; a multicloning site is located downstream of firefly luciferase. A fragment sequence of 454 bp was chemically synthesized containing either wild type human Foxo1 3′UTR (NM_002015) or the counterpart with mutations on miR-183-5p binding site (GTGCCAT), followed by the ligation into GV272, resulting in plasmid GV-Foxo1-3′UTR-WT or GV-Foxo1-3′UTR-Mut. To develop plasmid GV-hmiR-183-5p, a chemically synthesized DNA sequence was inserted into plasmid GV251, in which miR-183-5p will be translated under the promoter of human U6. All constructs were verified by sequencing. Renilla luciferase-expressing plasmid was ordered from Shanghai Genechem Company.

To verify the functional inhibition of miR-183 to human Foxo1 expression, dual-luciferase reporter assay was completed by cotransfection of miRNA plasmid (GV251 or GV-hmiR-183) and firefly luciferase plasmid (GV272, or GV-Foxo1-3′UTR-WT, or GV-Foxo1-3′UTR-WT). Briefly, 293T cells were seeded into a 24-well plate in 1 mL complete medium. The next day, miRNA plasmid (0.4 *μ*g for each) was cotransfected with firefly luciferase plasmid (0.1 *μ*g for each). In each transfection, Renilla luciferase-expressing plasmid (0.02 *μ*g for each) was incorporated as the internal control. After 48 hours, the cells were lysed and dual-luciferase assay was performed based on the instruction of Promega kit (Cat# E1910). The firefly luciferase reading was corrected by Renilla luciferase. The data were presented by relative luciferase activity as the mean ± standard deviation (SD).

### 2.9. Statistical Analysis

SPSS 23.0 (SPSS, USA) and GraphPad Prism 7.0 (GraphPad Software Inc., USA) were employed for data analysis. Data are presented as the mean ± SD, from triplicate assays repeated at least thrice. Two-tailed Student's *t*-test or one-way analysis of variance (ANOVA) was performed for comparisons. The Spearman's test was carried out to assess associations of candidate miRNAs with continuous variables in SLE cases. Receiver operating characteristic (ROC) curve analysis was carried out to evaluate the performances of candidate miRNAs and to distinguish SLE cases from controls. *p* < 0.05 indicated statistical significance.

## 3. Results

### 3.1. Correlation Heat Map Analysis for Total miRNA Patterns

**Figure 1:**
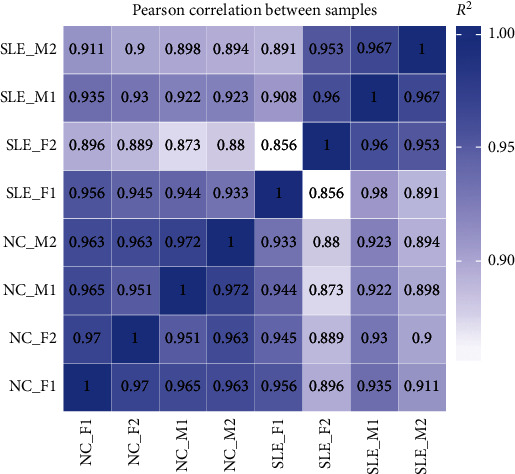
Correlation heat map analysis. SLE and controls presented distinct clusters based on the miRNA expression profiles of the eight specimens except for SLE_F1.

To investigate the distinct cluster of the miRNA expression profiles between the samples from SLE patients and healthy controls, Pearson correlation was performed. The correlation heat map demonstrated that SLE patients and controls had distinct clusters based on the miRNA expression profiles, except for SLE_F1 (Figure 1). Our data indicated that miRNA expression profiles were able to differentiate SLE patients from controls.

### 3.2. Volcano Plot and Heat Map Analyses

**Figure 2:**
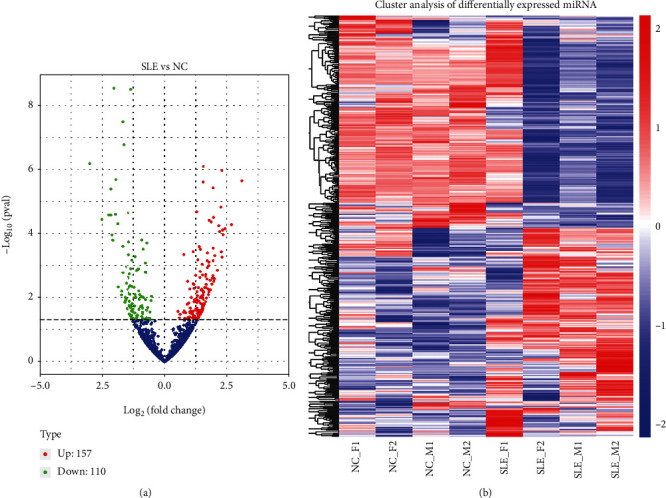
Volcano plot and heat map analyses. (a) Totally, 157 and 110 miRNAs were upregulated and downregulated, respectively, in SLE cases compared with controls. (b) The differentially expressed miRNAs well differentiated SLE cases from healthy controls.

To identify the differentially expressed miRNAs between SLE and controls, the hierarchical cluster analysis was carried out. The volcano plot revealed 157 upregulated and 110 downregulated miRNAs in SLE cases in comparison to controls [log2 (FC) > 0.5 and *p* < 0.05] (Figure 2(a)). The detailed information of 267 differentially expressed miRNAs is displayed in Supplementary Table 2. Heat map analysis revealed that these differentially expressed miRNAs could clearly discern SLE patients from controls (Figure 2(b)).

### 3.3. GO and KEGG Enrichment Analyses

**Figure 3:**
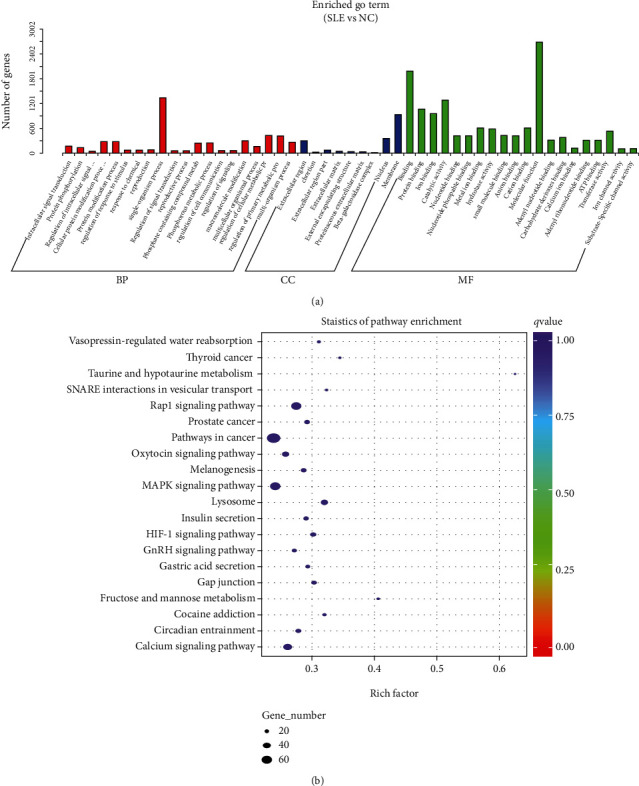
GO and KEGG enrichment analyses of the differentially expressed miRNAs. (a) GO enrichment analysis showed that the differentially expressed miRNAs were correlated with various biological processes such as molecular function, protein binding, and nucleotide binding activity. (b) KEGG enrichment analysis revealed the involvement of the differentially expressed miRNAs in various pathways such as cancer, MAPK signaling, and Rap1 signaling.

To assess the biological functions and pathways of the above differentially expressed miRNAs, GO and KEGG enrichment analyses were carried out. GO enrichment analysis revealed the involvement of the differentially expressed miRNAs in multiple biological processes such as molecular function, protein binding, and nucleotide binding activity (Figure 3(a)). KEGG enrichment analysis demonstrated that the differentially expressed miRNAs were involved in different pathways such as cancer, MAPK signaling, and Rap1 signaling (Figure 3(b)). Both enrichment analyses revealed that the differentially expressed miRNAs were implicated in inflammatory and immune activities.

### 3.4. Expression of Candidate miRNAs in SLE Cases and Healthy Controls in the Validation Phase

**Figure 4:**
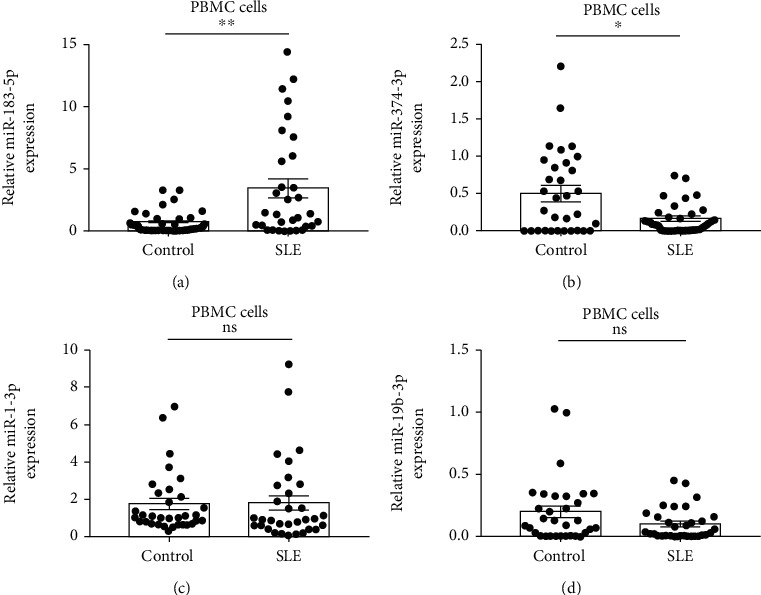
Expression levels of candidate miRNAs in SLE cases and controls in the validation phase. The expression level of miRNA was determined using qPCR from each group. Our data found that miR-183-5p was upregulated (a) and miR-374b-3p was downregulated (b). However, no significant difference was exhibited with regard to miR-1-3p (c) and miR-19b-3p (d). ns: *p* > 0.05; ^∗^*p* < 0.05; ^∗∗^*p* < 0.01.

To further evaluate miRNA dysregulation in SLE, two upregulated miRNAs (hsa-miR-1-3p and hsa-miR-183-5p) and two downregulated miRNAs (hsa-miR-374b-3p and hsa-miR-19b-3p) were assessed by qPCR in 32 SLE and 32 healthy controls. Among the four candidate miRNAs, miR-183-5p was upregulated (*p* = 0.005) (Figure 4(a)) and miR-374-3p was downregulated (*p* = 0.016) (Figure 4(b)) in SLE cases in comparison with controls, while miR-1-3p (*p* = 0.318) (Figure 4(c)) and miR-19b-3p (*p* = 0.115) (Figure 4(d)) levels did not show significance between the two groups.

### 3.5. Diagnostic Values of miRNAs

**Figure 5:**
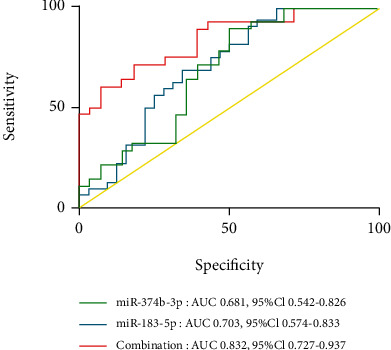
Diagnostic values of miR-183-5p and miR-374b-3p. ROC curve analysis indicated AUCs for miR-183-5p and miR-374b-3p of 0.703 (: 0.574–0.833) and 0.681 (95% CI: 0.542–0.82), respectively. Combination of miR-183-5p and miR-374b-3p yielded an AUC of 0.832 (95% CI: 0.727–0.937).

To evaluate the potential diagnostic value of miR-183-5p and miR-374b-3p in SLE, ROC curve analysis was performed. Areas under the ROC curves (AUCs) for miR-183-5p and miR-374b-3p were 0.703 (95% CI: 0.574–0.833) and 0.681 (95% CI: 0.542–0.826), respectively. Meanwhile, miR-183-5p combination with miR-374b-3p yielded an AUC of 0.832 (95% CI: 0.727–0.937) (Figure 5). These results suggested that miR-183-5p and/or miR-374b-3p presented a good diagnostic value for SLE detection, with the combination being superior to either miRNA used alone.

### 3.6. miR-183-5p Is Associated with Patient Data in SLE

**Figure 6:**
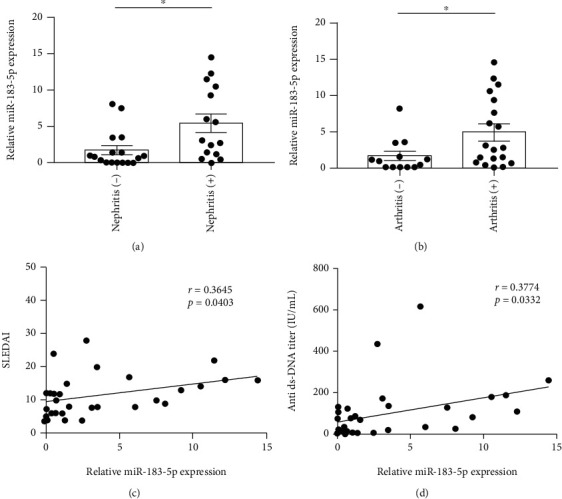
miR-183-5p is associated with patient data in SLE. The expression level of miR-183-5p was higher in SLE cases with nephritis (a) or arthritis (b) in comparison with negative SLE counterparts, showing significant positive associations with SLEDAI score (c) or anti-dsDNA antibody levels (d). ^∗^*p* < 0.05.

To determine the potential functions of miR-183-5p and miR-374b-3p in SLE, the associations of miR-183-5p and miR-374b-3p with the characteristics of SLE patients were examined. miR-183-5p was elevated in SLE cases with nephritis compared to the counterparts without nephritis (*p* = 0.018) (Figure 6(a)). SLE cases with arthritis had increased miR-183-5p amounts compared with counterparts without arthritis (*p* = 0.022) (Figure 6(b)). In correlation analysis, miR-183-5p expression displayed significant positive associations with SLEDAI score (*p* = 0.040) (Figure 6(c)) and anti-dsDNA antibody levels (*p* = 0.033) (Figure 6(d)). Meanwhile, miR-374b-3p amounts in SLE cases were similar regardless of clinical features (*p* >0.05), and no correlations of miR-374b-3p expression levels were observed with various clinical characteristics (*p* > 0.05).

### 3.7. Bioinformatics Analysis of the Potential Target Genes of miR-183-5p

**Figure 7:**
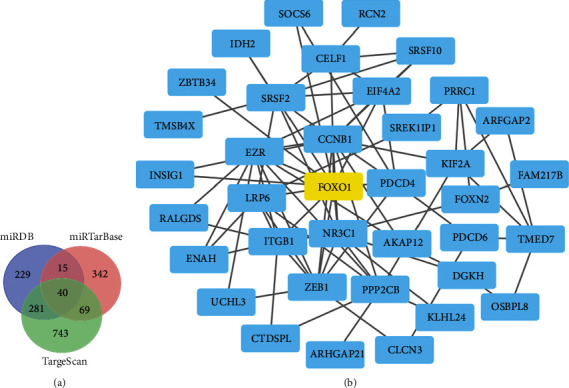
Bioinformatics analysis of the potential target genes of miR-183-5p. (a) The potential target genes of miR-183-5p were predicted in Targetscan7.1, miRDB, and miRTarBase platforms. (b) PPI network analysis showed the Foxo1 with high degrees of connectivity in potential target genes.

To further explore the underlying molecular mechanisms, the potential target genes of miR-183-5p were predicted in Targetscan (http://www.targetscan.org), miRDB (http://mirdb.org), and miRTarBase (http://mirtarbase.cuhk.edu.cn) platforms (Figure 7(a)). The overlapped genes on these three platforms were evaluated for Protein-Protein Interaction (PPI) network analysis to discover the interactions between the predictive target genes. Finally, the network consisting of 40 nodes and 81 edges is shown in Figure 7(b). Foxo1 was chosen for further investigations due to the high degree of connectivity.

### 3.8. Functional Binding of miR-183-5p to Foxo1

**Figure 8:**
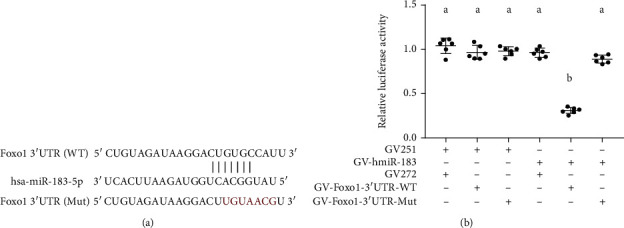
miR-183-5p directly targets Foxo1 3′UTR. (a) The binding site of miR-183-5p and the position 226-242 of Foxo1 3′UTR wild-type (WT) and mutant-type (mut). (b) miR-183-5p ectopic expression significantly inhibited luciferase activity of the wild-type Foxo1 3′UTR reporter plasmid in comparison with the mutated counterpart. Groups labeled with different letters are statistically different from each other. ^∗^*p* < 0.05. Differences between groups were analyzed for statistical significance by ANOVA with Fischer's probable least-square difference post hoc test.

To test whether miR-183-5p can directly target human Foxo1, we did bioinformatics analysis through the online tool, TargetScan platform (http://www.targetscan.org). We found miR-183-5p could directly bind to human Foxo1 3′UTR at sequences (GUGCCAU) (Figure 8(a)). To further very the binding in cell culture study, luciferase reporter plasmid containing Foxo1 3′UTR (and the mutant version) and miR-183-5p expressing plasmid were developed. The luciferase mRNA will be degraded once miR-183-5p binds to Foxo1 3′UTR. The plasmids were cotransfected into 293T cells for dual-luciferase assay. The data showed that the cotransfection of miR-183-5p plasmid with wild type 3′UTR luciferase plasmid knocked down luciferase activity significantly (Figure 8(b)), while the combination of miR-183-5p plasmid with mutant 3′UTR luciferase plasmid displayed a similar luciferase level to the mock transfections. Collectively, our cell culture study functionally demonstrated the targeting and binding of miR-183-5p to the wild type of human Foxo1 3′UTR rather than the mutant version.

### 3.9. Decreased mRNA Expression Levels of the Target Gene Foxo1

**Figure 9:**
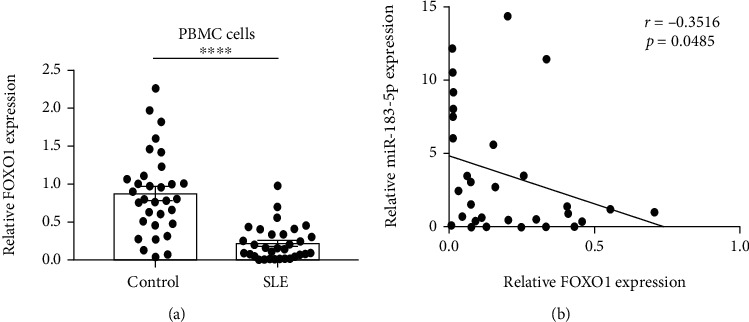
Expression of Foxo1 in subjects and with miR-183-5p levels (a) Foxo1 amounts are reduced in SLE cases (*n* = 32) in comparison with healthy controls (*n* = 32). (b) Spearman's rank correlation revealed a negative relationship between Foxo1 amounts and miR-183-5p expression levels. ^∗^*p* < 0.05; ^∗∗∗∗^*p* < 0.0001.

To answer the importance that miR-183-5p can target and bind to Foxo1 3′UTR in patients, we compared the expression level of Foxo1 between SLE cases and healthy controls and analyzed its correlation with miR-183-5p amounts in SLE. The Foxo1 mRNA expression level was markedly decreased in SLE cases compared with healthy controls (Figure 9, *p* < 0.0001). In correlation analysis, miR-183-5p and Foxo1 showed the inverse correlation in SLE patients (*p* = 0.049). The results further indicated that miR-183-5p is involved in the mechanisms of SLE by inhibiting the expression of Foxo1.

## 4. Discussion

Growing evidence indicates that miRNAs have critical functions in immune homeostasis and are involved in the pathophysiological mechanisms of various autoimmune ailments such as SLE [28–30]. Next-generation sequencing is capable of identifying novel transcripts and detecting low-expression transcripts. Here, NGS was performed to analyze the miRNA profiles of PBMCs from SLE and healthy controls. A total of 157 and 110 miRNAs were upregulated and downregulated, respectively, in SLE cases in comparison with healthy controls. GO enrichment analysis revealed the involvement of the differentially expressed miRNAs in various biological processes such as molecular function, protein binding, and nucleotide binding activity. In addition, KEGG analysis demonstrated that the target genes of differentially expressed miRNAs participated in the MAPK and Rap1 pathways, which regulate inflammatory responses.

The complex manifestations of SLE make its diagnosis difficult. Therefore, further assessment of the above miRNAs might help discover new diagnostic biomarkers of SLE. To this end, two upregulated and two downregulated miRNAs were selected and validated in 32 SLE cases and 32 controls. As depicted above, miR-183-5p and miR-374b-3p amounts were elevated and reduced, respectively, in SLE cases in comparison with controls. Meanwhile, the AUC for miR-183-5p combined with miR-374b-3p was 0.832 (95% CI: 0.727–0.937). These results demonstrated the diagnostic value of miR-183-5p and miR-374b-3p combination in SLE. Moreover, SLE cases with nephritis and arthritis had elevated miR-183-5p amounts compared with counterparts without these clinical features, indicating miR-183-5p might be involved in the destruction of the kidneys and joints. Next, the associations of miR-183-5p and miR-374b-3p with clinical characteristics of SLE cases were examined. As demonstrated above, miR-183-5p amounts displayed positive correlations with SLEDAI and anti-dsDNA antibody in SLE patients, indicating miR-183-5p could be a good indicator for evaluating SLE activity.

miR-374b was previously found to inhibit cell growth and promote apoptosis in T cell lymphoblastic lymphoma via suppression of AKT1 and Wnt-16 [31], which were correlated with immune activities in autoimmune diseases [32, 33]. In addition, miR-374b inhibits cell proliferation and enhances apoptosis via p38/ERK signaling by interacting with JAM-2 [34], suggesting miR-374b contributes to inflammation-associated pathways. Therefore, miR-374b may be involved in SLE-related inflammatory reactions.

miR-183-5p represents the main member of the miR-183 cluster, which can be dramatically induced in immune cells after activation. Previous reports indicated that the miR-183 cluster has a critical function in immune cell functions by regulating several proinflammatory pathways [35]. Thiel et al. found that miR-183 and miR-96 amounts are elevated in CD4+ T cells obtained from the peripheral blood of Graves' orbitopathy (GO) cases, and adoptive transfer of miR-183 and miR-96 overexpressing antigen-specific T cells accelerates the onset of autoimmune diabetes, whereas transferring specific antagomirs in CD4+ T cells prolongs disease onset [36]. miRNAs perform biological functions by inhibiting their target genes. We found Foxo1 was the potential functional target genes of miR-183-5p by bioinformatics analysis, and the direct targeting relationship was validated by dual-luciferase reporter assay in our study. Foxo1 activation is critical in autoimmune diseases [37, 38]. Furthermore, Foxo1 is also tightly correlated with the immune response. As shown above, Foxo1 amounts were significantly reduced in PBMCs from SLE cases in comparison with healthy controls, and miR-183-5p and Foxo1 showed an inverse correlation in SLE patients. This could reflect a potential mechanism wherein suppression of Foxo1 by miR-183-5p contributes to SLE pathogenesis. Ichiyama and colleagues reported that Foxo1 downregulation by miR-183 cluster constitutes one of the important mechanisms by which Th17 cells become pathogenic and induce disrupted balance between Treg and Th17 cells [39]. This further indicated that exploring the molecular mechanisms of miR-183-5p in SLE progression provides new insights into SLE etiology and could help identify novel therapeutic targets.

This study had several limitations. Firstly, the sample size was relatively small, and larger trials are warranted for the validation of these findings. Secondly, other autoimmune diseases should be assessed to confirm the specificities and sensitivities of these biomarkers. Finally, further functional studies are required to clarify the mechanism of these miRNAs in SLE.

## 5. Conclusion

miRNA expression profiling in PBMCs from SLE cases was significantly altered in comparison with healthy controls. We identified miR-183-5p as a potential diagnostic biomarker of SLE. miR-183-5p amounts showed positive associations with SLEDAI and anti-dsDNA antibody, implying that miR-183-5p is linked to SLE disease activity. Meanwhile, Foxo1, a miR-183-5p target, was markedly downregulated in SLE cases, indicating that miR-183-5p regulates the pathogenetic mechanisms and activity of SLE by inhibiting the expression of Foxo1. Further studies are required to uncover the functions of these miRNAs in SLE, which would eventually improve the diagnosis and treatment of SLE.

## Figures and Tables

**Table 1:** Clinicopathological and laboratory data of SLE and control cases.

Parameters	SLE (*n* = 32)	Healthy controls (*n* = 32)
Age (years)	34.8 ± 10.6	33.5 ± 10.3
Gender (male/female), *n*	4/28	4/28
Arthritis, *n* (%)	19 (59.4%)	—
Lupus nephritis, *n* (%)	15 (46.9%)	—
Serositis, *n* (%)	10 (31.3%)	—
Rash, *n* (%)	17 (53.1%)	—
ANA, *n* (%)	32 (100%)	—
ESR (mm/h)	39.83 ± 32.45	—
CRP (mg/L)	18.57 ± 28.31	—
Complement 3 (g/L)	0.71 ± 0.29	—
Anti-dsDNA antibody (IU/mL)	98.84 ± 130.46	—
SLEDAI score	11.34 ± 5.97	—

The average data of age, ESR, CRP, complement 3, anti-dsDNA, and SLEDAI score are presented as the mean ± SD.

## Data Availability

The data generated or analyzed in this study are available from the corresponding author upon reasonable request.

## Supplementary Materials

Supplementary 1 Supplementary Table 1: reverse transcription and PCR primer sequences.

Supplementary 2 Supplementary Table 2: the differentially expressed miRNAs between SLE cases and healthy controls.
