# Corrigendum to “miR-183-5p Is a Potential Molecular Marker of Systemic Lupus Erythematosus”

**DOI:** 10.1155/2021/9818203

**Published:** 2021-09-04

**Authors:** Shaolan Zhou, Jing Zhang, Pengfei Luan, Zhanbing Ma, Jie Dang, Hong Zhu, Qian Ma, Yanfeng Wang, Zhenghao Huo

**Affiliations:** ^1^Department of Medical Genetics and Cell Biology, College of Basic Medicine, Ningxia Medical University, Yinchuan, Ningxia, China; ^2^Department of Rheumatology, General Hospital of Ningxia Medical University, Yinchuan, Ningxia, China; ^3^Key Laboratory of Fertility Preservation and Maintenance (Ningxia Medical University), Ministry of Education, Yinchuan, Ningxia, China; ^4^Ningxia Key Laboratory of Cerebrocranial Diseases, Ningxia Medical University, Yinchuan, Ningxia, China; ^5^Department of Biology, Gansu Medical College, Pingliang, Gansu, China

In the article titled “miR-183-5p Is a Potential Molecular Marker of Systemic Lupus Erythematosus” [1], Figure 8 was formatted incorrectly. The authors have corrected this error and provided the correct figure as follows:

## Figures and Tables

**Figure 1:**
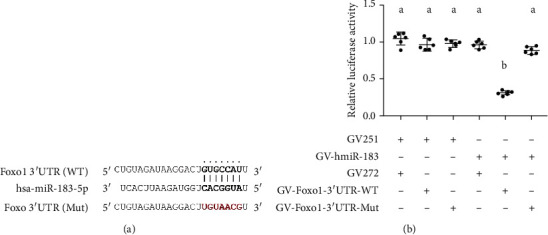
miR-183-5p directly targets Foxo1 3′UTR. (a) The binding site of miR-183-5p and the position 226-242 of Foxo1 3′UTR wild type (WT) and mutant type (mut). (b) miR-183-5p ectopic expression significantly inhibited luciferase activity of the wild-type Foxo1 3′UTR reporter plasmid in comparison with the mutated counterpart. Groups labelled with different letters are statistically different from each other (^∗^*p* < 0.05). Differences between groups were analyzed for statistical significance by ANOVA with Fischer's probable least-square difference post hoc test.
