# Supplementary material for: miR-183-5p Is a Potential Molecular Marker of Systemic Lupus Erythematosus
Source: J Immunol Res. 2021 May 6;2021:5547635. doi: 10.1155/2021/5547635 (PMC8124875; doi:10.1155/2021/5547635)
Supplement: Supplementary 1 — Supplementary Table 1: reverse transcription and PCR primer sequences. [file 5547635.f1.pdf]

Supplementary Table 1. Reverse transcription gene specific primer and PCR primer sequences

PCR Target		Primer sequence (5'---3')
U6	Reverse ranscription primer	GTCGTATCCAGTGCAGGGTCCGAGGTATTCGCACTGGATACGACAAAATA
	Forward Primer	AGAGAAGATTAGCATGGCCCCTG
	Reverse primer	GTCGTATCCAGTGCAGGGTCCGAGGTATTCGCACTGGATACGACAAAATA
hsa-miR-1-3p	Reverse ranscription primer	GTCGTATCCAGTGCAGGGTCCGAGGTATTCGCACTGGATACGACATACAT
	Forward Primer	GCGCGTGGAATGTAAAGAAGT
	Reverse primer	AGTGCAGGGTCCGAGGTATT
hsa-miR-183-5p	Reverse ranscription primer	GTCGTATCCAGTGCAGGGTCCGAGGTATTCGCACTGGATACGACAGTGAA
	Forward Primer	CGCGTATGGCACTGGTAGAA
	Reverse primer	AGTGCAGGGTCCGAGGTATT
hsa-miR-374b-3p	Reverse ranscription primer	GTCGTATCCAGTGCAGGGTCCGAGGTATTCGCACTGGATACGACAATGAT
	Forward Primer	CGCGCTTAGCAGGTTGTATT
	Reverse primer	AGTGCAGGGTCCGAGGTATT
hsa-miR-19b-3p	Reverse ranscription primer	GTCGTATCCAGTGCAGGGTCCGAGGTATTCGCACTGGATACGACTCAGTT
	Forward Primer	CGTGTGCAAATCCATGCAA
	Reverse primer	AGTGCAGGGTCCGAGGTATT
Gapdh	Forward Primer	CAGGAGGCATTGCTGATGAT
	Reverse primer	GAAGGCTGGGGCTCATTT
Foxo1	Forward Primer	CTACGAGTGGATGGTCAAGAGC
	Reverse primer	CAGTTCCTTCATTCTGCACACG
