# Supplementary material for: miR-183-5p Is a Potential Molecular Marker of Systemic Lupus Erythematosus
Source: J Immunol Res. 2021 May 6;2021:5547635. doi: 10.1155/2021/5547635 (PMC8124875; doi:10.1155/2021/5547635)
Supplement: Supplementary 2 — Supplementary Table 2: the differentially expressed miRNAs between SLE cases and healthy controls. [file 5547635.f2.pdf]

Supplementary Table 2. The differentially expressed miRNAs between SLE cases and healthy controls.

| Gene symbol | LogFC | P value | Trend |
|---|---|---|---|
| hsa-miR-4697-5p | 3.0761 | 2.25E-06 | up |
| hsa-miR-6506-5p | 2.663 | 5.21E-05 | up |
| **hsa-miR-1-3p** | **2.4092** | **6.92E-05** | **up** |
| hsa-miR-412-5p | 2.3383 | 0.00010739 | up |
| hsa-miR-379-5p | 2.3172 | 7.67E-05 | up |
| hsa-miR-6716-3p | 2.2924 | 0.00053429 | up |
| hsa-miR-495-3p | 2.2832 | 1.01E-06 | up |
| hsa-miR-380-5p | 2.2633 | 0.00036412 | up |
| hsa-miR-432-5p | 2.2494 | 1.48E-05 | up |
| hsa-miR-206 | 2.1954 | 8.54E-05 | up |
| hsa-miR-369-5p | 2.1623 | 5.59E-05 | up |
| hsa-miR-937-3p | 2.1081 | 0.001374 | up |
| hsa-miR-204-5p | 2.0035 | 0.00047165 | up |
| hsa-miR-4697-3p | 1.9944 | 0.0025636 | up |
| novel_833 | 1.9904 | 0.0025384 | up |
| novel_627 | 1.9629 | 0.0030856 | up |
| hsa-miR-1180-3p | 1.9548 | 3.09E-05 | up |
| hsa-miR-4746-5p | 1.9449 | 0.0006899 | up |
| hsa-miR-6741-3p | 1.9425 | 0.00028075 | up |
| hsa-miR-139-5p | 1.9393 | 3.74E-06 | up |
| novel_759 | 1.9346 | 0.0020498 | up |
| hsa-miR-2115-3p | 1.9088 | 0.0024564 | up |
| novel_780 | 1.8961 | 0.0037641 | up |
| hsa-miR-143-3p | 1.8657 | 4.27E-05 | up |
| hsa-miR-5584-5p | 1.8319 | 0.0056851 | up |
| hsa-miR-493-3p | 1.824 | 0.0029023 | up |
| hsa-miR-3678-3p | 1.8094 | 0.0055666 | up |
| hsa-miR-3614-5p | 1.7822 | 3.82E-05 | up |
| hsa-miR-134-5p | 1.7809 | 0.00076475 | up |
| hsa-miR-3146 | 1.7758 | 0.0065214 | up |
| hsa-miR-451a | 1.767 | 0.00097451 | up |
| hsa-miR-6843-3p | 1.7537 | 0.0080842 | up |
| hsa-miR-409-3p | 1.7369 | 0.0053542 | up |
| hsa-miR-182-5p | 1.7144 | 0.00039123 | up |
| hsa-miR-4661-5p | 1.7111 | 0.0011199 | up |
| hsa-miR-411-3p | 1.7069 | 0.00069136 | up |
| hsa-miR-214-3p | 1.6976 | 0.002638 | up |
| hsa-miR-4800-5p | 1.6764 | 0.01029 | up |
| **hsa-miR-183-5p** | **1.6672** | **0.0006478** | **up** |

| | | | |
|---|---|---|---|
| hsa-miR-1288-3p | 1.6649 | 0.0038882 | up |
| hsa-miR-1255b-5p | 1.6548 | 0.0048867 | up |
| hsa-miR-4504 | 1.6505 | 0.0018967 | up |
| hsa-miR-3120-5p | 1.6343 | 0.013532 | up |
| hsa-miR-6772-3p | 1.628 | 0.0021791 | up |
| hsa-miR-382-3p | 1.5871 | 0.0047292 | up |
| novel_534 | 1.5865 | 0.016058 | up |
| hsa-miR-382-5p | 1.5646 | 0.0066984 | up |
| hsa-miR-3662 | 1.5644 | 0.017107 | up |
| novel_517 | 1.555 | 0.016911 | up |
| hsa-miR-4437 | 1.5502 | 0.016472 | up |
| novel_801 | 1.5462 | 0.0061826 | up |
| hsa-miR-1268a | 1.5408 | 0.015114 | up |
| novel_259 | 1.5395 | 0.011104 | up |
| hsa-miR-625-3p | 1.5383 | 7.62E-07 | up |
| hsa-miR-379-3p | 1.5372 | 0.006662 | up |
| hsa-miR-654-5p | 1.5269 | 0.007988 | up |
| hsa-miR-5010-3p | 1.526 | 2.38E-06 | up |
| hsa-miR-3127-5p | 1.5228 | 0.021038 | up |
| hsa-miR-370-3p | 1.5095 | 0.016431 | up |
| hsa-miR-3605-3p | 1.5055 | 0.0084374 | up |
| hsa-miR-139-3p | 1.5031 | 0.0019284 | up |
| hsa-miR-6819-5p | 1.4917 | 0.021975 | up |
| hsa-miR-1299 | 1.4912 | 0.017292 | up |
| novel_220 | 1.4894 | 0.024725 | up |
| hsa-miR-375 | 1.4893 | 0.0057839 | up |
| hsa-miR-2276-3p | 1.4868 | 0.020835 | up |
| hsa-miR-486-3p | 1.4834 | 0.00088779 | up |
| hsa-miR-4800-3p | 1.4735 | 0.023649 | up |
| hsa-miR-148a-3p | 1.4686 | 0.0099877 | up |
| hsa-miR-431-5p | 1.4683 | 0.0053334 | up |
| hsa-miR-6842-3p | 1.4634 | 0.0089197 | up |
| hsa-miR-4657 | 1.4599 | 0.027462 | up |
| hsa-miR-3149 | 1.4434 | 0.027365 | up |
| hsa-miR-100-5p | 1.4425 | 0.0057074 | up |
| novel_770 | 1.4224 | 0.0147 | up |
| hsa-miR-4684-3p | 1.4197 | 0.032357 | up |
| hsa-miR-4687-5p | 1.4182 | 0.027615 | up |
| hsa-miR-23a-5p | 1.412 | 0.00030553 | up |
| hsa-miR-486-5p | 1.4077 | 0.0014919 | up |
| hsa-miR-6514-5p | 1.4058 | 0.0096785 | up |
| hsa-miR-205-5p | 1.4048 | 0.02513 | up |
| novel_484 | 1.4 | 0.030091 | up |
| hsa-miR-3120-3p | 1.3967 | 0.0023144 | up |

| | | | |
|---|---|---|---|
| hsa-miR-3939 | 1.3875 | 0.030522 | up |
| hsa-miR-6763-5p | 1.3836 | 0.030956 | up |
| hsa-miR-5189-5p | 1.3751 | 0.033262 | up |
| hsa-miR-126-3p | 1.3721 | 0.00025338 | up |
| hsa-miR-4533 | 1.3659 | 0.03942 | up |
| hsa-miR-337-3p | 1.3555 | 0.023986 | up |
| hsa-miR-1292-5p | 1.3473 | 0.012388 | up |
| hsa-miR-541-3p | 1.341 | 0.037318 | up |
| hsa-miR-4738-3p | 1.3394 | 0.032581 | up |
| novel_958 | 1.3391 | 0.033506 | up |
| hsa-miR-214-5p | 1.3361 | 0.0048002 | up |
| hsa-miR-933 | 1.3304 | 0.042896 | up |
| hsa-miR-539-5p | 1.3155 | 0.024079 | up |
| hsa-miR-323a-3p | 1.3147 | 0.02691 | up |
| hsa-miR-574-5p | 1.3116 | 0.00064466 | up |
| hsa-miR-4664-3p | 1.305 | 0.030163 | up |
| hsa-miR-556-5p | 1.2958 | 0.0084701 | up |
| hsa-miR-589-5p | 1.2933 | 0.012687 | up |
| hsa-miR-6847-5p | 1.2903 | 0.048446 | up |
| hsa-miR-1285-5p | 1.2873 | 0.013976 | up |
| hsa-miR-758-3p | 1.2848 | 0.021047 | up |
| hsa-miR-584-5p | 1.2804 | 0.011756 | up |
| hsa-miR-503-5p | 1.2801 | 0.0063908 | up |
| hsa-miR-30a-3p | 1.2691 | 2.04E-05 | up |
| hsa-miR-1908-5p | 1.2497 | 0.010941 | up |
| hsa-miR-4482-5p | 1.2463 | 0.043173 | up |
| hsa-miR-3158-5p | 1.2459 | 0.021966 | up |
| hsa-miR-4435 | 1.2456 | 0.044014 | up |
| hsa-miR-3158-3p | 1.2442 | 0.021854 | up |
| hsa-miR-6859-5p | 1.2376 | 0.023742 | up |
| hsa-miR-3615 | 1.2255 | 0.0019897 | up |
| hsa-miR-4786-5p | 1.2117 | 0.047767 | up |
| hsa-miR-6502-5p | 1.21 | 0.045437 | up |
| hsa-miR-3191-5p | 1.1944 | 0.037096 | up |
| hsa-miR-20b-3p | 1.1925 | 0.038081 | up |
| hsa-miR-576-3p | 1.1797 | 0.011555 | up |
| hsa-miR-1246 | 1.1706 | 0.013248 | up |
| hsa-miR-99b-5p | 1.1687 | 0.0050675 | up |
| hsa-miR-3190-3p | 1.1637 | 0.043388 | up |
| hsa-miR-5091 | 1.1588 | 0.0038313 | up |
| hsa-miR-543 | 1.1581 | 0.037677 | up |
| hsa-miR-889-3p | 1.1422 | 0.041762 | up |
| hsa-miR-219a-1-3p | 1.137 | 0.035462 | up |
| hsa-miR-10b-5p | 1.1325 | 0.00036078 | up |

| | | | |
|---|---|---|---|
| hsa-miR-3150a-5p | 1.1299 | 0.025771 | up |
| hsa-miR-411-5p | 1.1285 | 0.0036291 | up |
| hsa-miR-6877-5p | 1.1086 | 0.043187 | up |
| hsa-miR-1306-3p | 1.0944 | 0.03155 | up |
| hsa-miR-654-3p | 1.0894 | 0.041839 | up |
| hsa-miR-151b | 1.06 | 0.0096479 | up |
| hsa-miR-3913-5p | 1.0318 | 0.025841 | up |
| hsa-miR-3913-3p | 1.0288 | 0.029592 | up |
| hsa-miR-7705 | 1.0114 | 0.020578 | up |
| hsa-miR-504-5p | 0.99894 | 0.028827 | up |
| hsa-miR-15b-3p | 0.99819 | 0.0052693 | up |
| hsa-miR-664a-5p | 0.99386 | 0.027686 | up |
| hsa-miR-18a-3p | 0.98943 | 0.038704 | up |
| hsa-miR-3928-3p | 0.97215 | 0.044479 | up |
| hsa-miR-26a-2-3p | 0.95394 | 0.014361 | up |
| hsa-miR-148a-5p | 0.93647 | 0.040556 | up |
| hsa-miR-425-5p | 0.92362 | 0.04078 | up |
| hsa-miR-760 | 0.90976 | 0.047854 | up |
| hsa-miR-1273h-5p | 0.90598 | 0.0030486 | up |
| hsa-miR-6515-5p | 0.89441 | 0.015817 | up |
| hsa-miR-151a-5p | 0.82806 | 0.028904 | up |
| hsa-miR-671-3p | 0.82365 | 0.045981 | up |
| hsa-miR-363-5p | 0.80054 | 0.047557 | up |
| hsa-miR-10a-5p | 0.79856 | 0.045068 | up |
| hsa-miR-625-5p | 0.77613 | 0.025876 | up |
| hsa-miR-223-5p | 0.75744 | 0.00044765 | up |
| hsa-miR-193a-5p | 0.75152 | 0.028417 | up |
| hsa-miR-3074-5p | 0.61534 | 0.032333 | up |
| hsa-miR-24-3p | 0.60922 | 0.032596 | up |
| hsa-miR-576-5p | 0.58467 | 0.042922 | up |
| hsa-miR-1277-3p | -2.9851 | 6.21E-07 | down |
| hsa-miR-615-3p | -2.5139 | 3.59E-05 | down |
| hsa-let-7f-2-3p | -2.2449 | 2.58E-05 | down |
| hsa-miR-1298-5p | -2.1663 | 4.04E-06 | down |
| hsa-miR-873-5p | -2.1616 | 2.61E-05 | down |
| hsa-miR-1277-5p | -2.1213 | 0.00010851 | down |
| novel_549 | -2.0885 | 0.00015769 | down |
| hsa-miR-181a-5p | -2.0258 | 2.92E-09 | down |
| hsa-miR-142-3p | -1.9841 | 2.46E-05 | down |
| hsa-miR-618 | -1.9662 | 2.05E-06 | down |
| hsa-miR-30e-5p | -1.892 | 4.87E-05 | down |
| hsa-miR-1537-5p | -1.8777 | 0.0046178 | down |
| hsa-miR-3684 | -1.7469 | 0.0081847 | down |
| hsa-miR-577 | -1.7397 | 0.0062577 | down |

| | | | |
|---|---|---|---|
| hsa-miR-181c-5p | -1.6947 | 0.00024709 | down |
| hsa-miR-3667-3p | -1.691 | 0.0016539 | down |
| hsa-let-7g-5p | -1.6642 | 3.16E-08 | down |
| hsa-miR-181d-5p | -1.6216 | 1.62E-07 | down |
| novel_599 | -1.6139 | 0.014069 | down |
| hsa-miR-1255a | -1.5964 | 0.0097857 | down |
| hsa-miR-590-5p | -1.5909 | 0.012219 | down |
| hsa-miR-21-3p | -1.5484 | 0.003558 | down |
| hsa-miR-95-3p | -1.5481 | 0.0010592 | down |
| hsa-miR-3611 | -1.5348 | 0.010645 | down |
| hsa-miR-455-3p | -1.5211 | 0.021811 | down |
| hsa-miR-219a-5p | -1.4902 | 0.022764 | down |
| hsa-miR-660-5p | -1.4891 | 0.0055126 | down |
| **hsa-miR-374b-3p** | **-1.4661** | **2.20E-05** | **down** |
| hsa-miR-652-5p | -1.4621 | 0.002227 | down |
| hsa-miR-212-5p | -1.453 | 0.0024052 | down |
| hsa-miR-181b-2-3p | -1.446 | 0.0070843 | down |
| hsa-miR-340-5p | -1.4436 | 0.0047164 | down |
| hsa-miR-301a-5p | -1.4312 | 0.00045011 | down |
| hsa-miR-26b-5p | -1.4282 | 0.00018009 | down |
| **hsa-miR-19b-3p** | **-1.4038** | **0.017248** | **down** |
| hsa-miR-219b-3p | -1.3972 | 0.031563 | down |
| hsa-miR-32-5p | -1.3954 | 0.012774 | down |
| hsa-miR-548k | -1.3939 | 0.0017977 | down |
| hsa-miR-4423-3p | -1.3913 | 0.032296 | down |
| hsa-miR-874-3p | -1.3803 | 0.019193 | down |
| novel_231 | -1.3733 | 0.035057 | down |
| hsa-miR-29a-3p | -1.3729 | 0.0077875 | down |
| hsa-miR-140-5p | -1.3653 | 0.0042145 | down |
| hsa-miR-192-5p | -1.3537 | 3.17E-09 | down |
| hsa-miR-16-1-3p | -1.3505 | 0.018135 | down |
| hsa-miR-31-5p | -1.3475 | 0.019449 | down |
| hsa-miR-3065-5p | -1.338 | 0.0012164 | down |
| hsa-miR-338-3p | -1.338 | 0.0012164 | down |
| hsa-miR-542-5p | -1.3236 | 0.043054 | down |
| hsa-miR-642b-3p | -1.3164 | 0.025439 | down |
| hsa-miR-548ab | -1.3144 | 0.017192 | down |
| hsa-miR-642a-5p | -1.3097 | 0.024931 | down |
| hsa-miR-125b-2-3p | -1.3078 | 0.0043735 | down |
| hsa-miR-548aw | -1.3039 | 0.0465 | down |
| hsa-miR-141-5p | -1.3034 | 0.043309 | down |
| novel_756 | -1.3029 | 0.040642 | down |
| hsa-miR-1537-3p | -1.2983 | 0.034944 | down |
| hsa-let-7a-3p | -1.2933 | 0.00066245 | down |

| | | | |
|---|---|---|---|
| hsa-miR-1303 | -1.2837 | 0.015068 | down |
| hsa-miR-144-3p | -1.2616 | 0.043562 | down |
| hsa-miR-548a-3p | -1.2584 | 0.022106 | down |
| hsa-miR-1271-5p | -1.2537 | 0.027231 | down |
| hsa-miR-20a-5p | -1.2175 | 0.010021 | down |
| hsa-miR-598-3p | -1.2085 | 0.004661 | down |
| hsa-miR-3157-3p | -1.1813 | 0.017789 | down |
| hsa-miR-33a-3p | -1.1751 | 0.029496 | down |
| hsa-miR-627-3p | -1.1737 | 0.0079341 | down |
| hsa-miR-199b-5p | -1.1692 | 0.00051815 | down |
| hsa-miR-6718-5p | -1.137 | 0.0096087 | down |
| hsa-miR-20a-3p | -1.134 | 0.046272 | down |
| hsa-miR-362-5p | -1.1146 | 0.0014019 | down |
| hsa-miR-548s | -1.1102 | 0.049024 | down |
| hsa-miR-125a-5p | -1.0988 | 0.036089 | down |
| hsa-miR-301b-3p | -1.0967 | 0.041536 | down |
| hsa-miR-9-3p | -1.0896 | 0.037808 | down |
| hsa-miR-301a-3p | -1.0734 | 0.034566 | down |
| hsa-miR-505-3p | -1.0486 | 0.001275 | down |
| hsa-miR-181b-5p | -1.0347 | 0.0012366 | down |
| hsa-miR-2277-5p | -1.027 | 0.0060647 | down |
| hsa-miR-19a-3p | -1.0171 | 0.022582 | down |
| hsa-miR-130a-3p | -1.001 | 0.011128 | down |
| hsa-miR-148b-3p | -0.98675 | 0.0079676 | down |
| hsa-miR-580-3p | -0.961 | 0.018714 | down |
| hsa-miR-181c-3p | -0.91762 | 0.0092855 | down |
| hsa-miR-4772-5p | -0.91063 | 0.038119 | down |
| hsa-miR-30c-5p | -0.90896 | 0.0091592 | down |
| hsa-miR-361-3p | -0.90421 | 0.00015885 | down |
| hsa-miR-15b-5p | -0.8952 | 0.017393 | down |
| hsa-miR-181a-2-3p | -0.87877 | 0.027729 | down |
| hsa-miR-7-1-3p | -0.87305 | 0.020837 | down |
| hsa-miR-199a-5p | -0.86837 | 0.030856 | down |
| hsa-miR-1291 | -0.86655 | 0.045496 | down |
| hsa-miR-378a-5p | -0.85911 | 0.04904 | down |
| hsa-miR-146a-5p | -0.84962 | 0.00030782 | down |
| hsa-miR-26a-5p | -0.83839 | 0.0096068 | down |
| hsa-miR-1278 | -0.79577 | 0.04673 | down |
| hsa-miR-103b | -0.76155 | 0.0015903 | down |
| hsa-miR-9-5p | -0.75947 | 0.00083226 | down |
| hsa-let-7i-5p | -0.75666 | 0.011236 | down |
| hsa-miR-103a-3p | -0.73966 | 0.0016107 | down |
| hsa-miR-671-5p | -0.71945 | 0.04984 | down |
| hsa-miR-30d-3p | -0.7128 | 0.024029 | down |

| | | | |
|---|---|---|---|
| hsa-miR-30d-5p | -0.70922 | 0.041816 | down |
| hsa-let-7f-5p | -0.70134 | 0.00019829 | down |
| hsa-miR-500a-3p | -0.66932 | 0.019784 | down |
| hsa-miR-1287-5p | -0.60406 | 0.049927 | down |
| hsa-miR-532-5p | -0.58976 | 0.044563 | down |
| hsa-miR-484 | -0.57774 | 0.027497 | down |
| hsa-let-7a-5p | -0.53613 | 0.012094 | down |
| hsa-miR-146b-5p | -0.52286 | 0.0088433 | down |
